# The Role of *Typha angustifilia* Fiber–Matrix Bonding Parameters on Interfacial Shear Strength Analysis

**DOI:** 10.3390/polym14051006

**Published:** 2022-03-02

**Authors:** Syifaul Huzni, Ikramullah Ikramullah, Israr B. M. Ibrahim, Syarizal Fonna, Teuku Arriessa Sukhairi, Andri Afrizal, Umar Muksin, Abdul Khalil H. P. S., Sri Aprilia, Samsul Rizal

**Affiliations:** 1 Department of Mechanical and Industrial Engineering, Universitas Syiah Kuala, Darussalam, Banda Aceh 23111, Indonesia; ikramullah@unsyiah.ac.id (I.I.); israr@unsyiah.ac.id (I.B.M.I.); syarizal.fonna@unsyiah.ac.id (S.F.); teuku.arriessa@unsyiah.ac.id (T.A.S.); andri85@mhs.unsyiah.ac.id (A.A.); 2 Department of Physics, Universitas Syiah Kuala, Darussalam, Banda Aceh 23111, Indonesia; muksin.umar@unsyiah.ac.id; 3 School of Industrial Technology, Universiti Sains Malaysia, Penang 11800, Malaysia; akhalilhps@gmail.com; 4 Department of Chemical Engineering, Universitas Syiah Kuala, Darussalam, Banda Aceh 23111, Indonesia; sriaprilia@unsyiah.ac.id

**Keywords:** microbond, *Typha angustifolia*, interfacial fiber–matrix bonding, finite element method

## Abstract

The microbond test of natural fibers tends to produce scattered interfacial shear stress (IFSS) values. The sources of this scattering are known, but the roles they play in producing high IFSS scattering remain to be investigated. In this study, a numerical method was used to simulate microbond testing and to examine the experimental parameters in a microbond test of *Typha angustifolia* fiber/epoxy. Three parameters were considered: fiber diameter, fiber length embedded in the epoxy, and the distance between the vise and the specimen. The geometries were modeled and analyzed by ABAQUS software using its cohesive zone model features. There were two types of contact used in this analysis: tie constraint and surface-to-surface. The results showcased the roles of the following experimental parameters: a larger fiber diameter from a sample increased the IFSS value, a longer embedded length reduced the IFSS value, and a shorter vise–specimen distance increased the IFSS value. The IFSS scattering in the microbond test could have originated from the interaction between these parameters. Of the three parameters, only the vise–specimen distance was found to be able to be reasonably controlled. When the IFSS value was atypically large, fiber diameter and/or embedded length potentially drove the scattering. This study advises further compilation and classification of the role of each experimental parameter in modulating the IFSS value.

## 1. Introduction

Many types of natural fibers are abundantly available in nature—one of them is *Typha angustifolia* fiber. *Typha angustifolia* fiber has the potential to be a substitute for synthetic fibers in fiber-reinforced composites*. Typha angustifolia* plants are grown in most countries [1]. This plant is still considered a parasite, since its growth dominates in wetlands. Compared to other natural fibers, its potential has been unutilized [2,3]. In several previous studies, *Typha angustifolia* fiber was found to have several advantages such as excellent bending properties, light weight, excellent mechanical properties, low density, and renewability [4,5,6]. Even though natural fiber has many advantages, many researchers have highlighted the fact that the incompatibility between hydrophilic cellulose and hydrophobic polymer is a crucial weakness of natural fibers. Bonding natural fibers and polymers is a challenge due to the different chemical structures of fibers and matrices. The incompatibility between the fiber and the matrix reduces the interface bond performance between the fiber and the matrix. The interfacial bonding greatly affects the mechanical properties of composite material [7]. 

The interfacial shear strength (IFSS) of the fiber–matrix bond can be measured using several testing methods such as the microbond test, the pull-out test, the push-out test, and the fragmentation test. The microbond test method developed by Miller et al. [8] is one of the most widely used test methods for the examination of the IFSS of a fiber–matrix bond [9]. These tests are difficult to conduct experimentally [10] for several reasons, such as the difficulty in accurately measuring the length of the fiber embedded in the matrix due to the formation of the meniscus at both ends of the matrix as well as difficulty in measuring the failure mechanism at the microscale level due to the very small size of the microdroplet [11]. On the other hand, it is often found that the fibers break before debonding occurs, requiring high accuracy and producing scattered results. Therefore, the numerical method approach has been used to simulate these tests with the finite element method to facilitate IFSS examination [12]. 

Two important issues affecting the fragmentation rate and the final critical fragment length are the axial stress profile in the fiber and the length of the transfer zone. In terms of a perfect plastic matrix that should be attached to the fiber, the stress formation in the fiber should be more linear, and the length of the stress transfer zone should be proportional to the fiber’s far-field stress. The partial-debonding model assumes that the stress of the fiber is non-linear due to the debonded region at the end of the fragment and the fact that stress transfer is associated with elastic friction and shear. The non-linearity of the stress distribution of the fiber has been confirmed experimentally, and stress transfer with a fiber fragment has generally been shown to include three regions, namely, debonded, bounded yielded, and bounded unyielded [13]. Therein, the stress concentration at the fiber break is reduced by fiber debonding and plastic deformation of the matrix. 

Zhi et al. [14] investigated the interface bonding properties of ternary composites with epoxy resin as a matrix, using micro balloons (microbubbles) as phase particles with reinforced polyester fibers. The 3D finite element model was created using ANSYS on the basis of contact analysis. The interface bonding properties of ternary micro balloon/epoxy composites with fiber reinforcement (FMETCs) were determined by differentiating fiber diameter parameters through the analysis of the size and content of the particles. 

Potukuchi [15] simulated microbond testing on carbon fiber-reinforced composites with an epoxy resin matrix. The modelling was presented in axisymmetric form with different surface treatment types on carbon fiber, identifying the cohesive failure properties of the interface for each case. The verification of the cohesive model was conducted by comparing the force (F) and the displacement (δ) that were obtained from the simulation results with those obtained experimentally; thereafter, a parametric study was conducted to check the stability and behavior of the cohesive failure model. Song et al. [16] developed a method of determining the correct contact angle for drop-on-fiber systems using digital image processing techniques in order to determine the shape of the matrix droplets. The contact angle of the extracted matrix droplet shape was obtained by the long-drop method. This method can more easily and reliably be used to calculate the contact angle occurring between fibers and matrices in comparison with the original maximum length derivation method. Pandey et al. [17] studied the effect of blade separation from fiber and blade geometry on the stress distribution in the interface plane of the fiber and epoxy resin matrix using the microbond test simulation method. They produced two axisymmetric models in 2D and one model in three dimensions (3D), and then made comparisons with the 3D model by taking into account the friction between the blade and the surface of the matrix drops alongside the results obtained in the 3D model, regardless of the friction that occurred. 

In the microbond test developed by Miller et al. [8], the pull-out was carried out on a composite specimen containing a single fiber and one resin drop; the authors reported that the fiber diameter and embedded length affected the IFSS value. In addition, the stress distribution was also affected by the vise–specimen distance [18]. Therefore, the effect of fiber diameter, embedded length, and vise–specimen distance needs to be studied. The objective of this study was to determine the effect of fiber diameter, embedded length, and the distance between the vise and the specimen on the *Typha angustifolia* fiber/epoxy microbond test using the finite element method. 

These parameters are a known source of data scattering in a microbond test. However, the way in which they influence microbond test results in a manner that leads to IFSS value scattering is difficult to determine experimentally since they are difficult to control and design in a microbond test. This study provides a numerical simulation solution to explain the relationship between these parameters and the IFSS value. To investigate IFSS scattering in a microbond test, one should compile and classify the role of each experimental parameter in modulating the IFSS value. The findings of this study will be a valuable addition to future investigations on IFSS scattering in a microbond test. 

## 2. Materials and Methods

**Figure 1:**
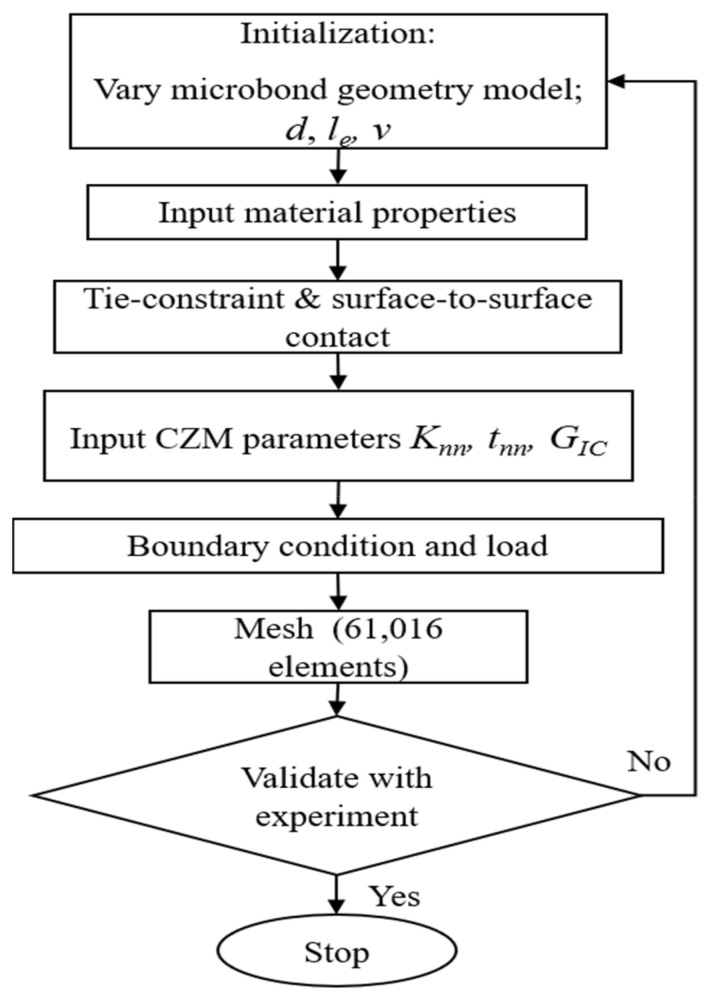
Flowchart of *Typha angustifolia* fiber/epoxy microbond test simulation.

The *Typha angustifolia* fiber/epoxy microbond test was previously carried out experimentally by Rizal et al. [18]; all of the microbond test geometry was modelled by the finite element method using the ABAQUS software. Figure 1 presents the simulation flowchart regarding the finite element method. 

**Figure 2:**
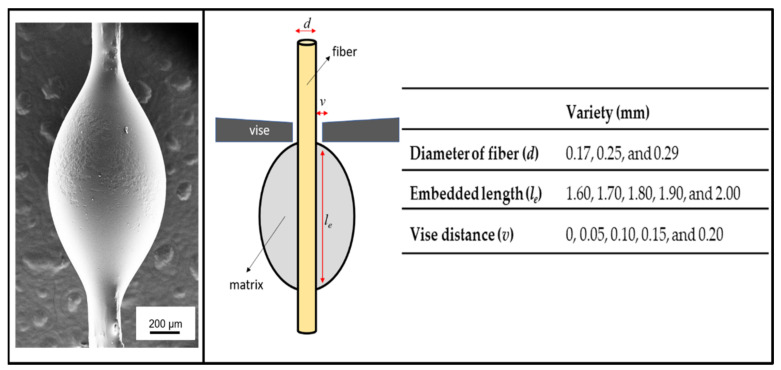
Left: SEM image of epoxy microdroplet of *Typha angustifolia* fiber. Middle: *Typha angustifolia* fiber/epoxy microbond test model. Right: The dimensions of the model. Each dimension parameter had several variations.

Figure 2 shows the geometry and various dimensions of the *Typha angustifolia* fiber/epoxy microbond test model used in this study. This simulation model was experimentally derived from the geometry of the microbond test.

The material properties of the *Typha angustifolia* fiber/epoxy microbond test model are presented in Table 1. 

### 2.1. Contact Model

**Figure 3:**
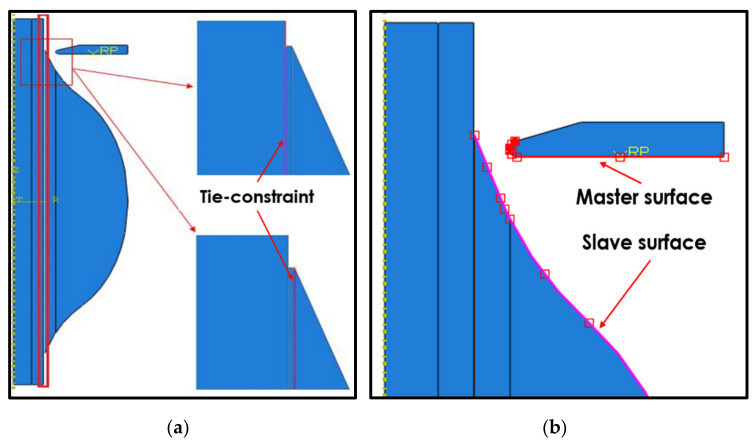
(**a**) The tie constraint of fiber contact with cohesive elements and a matrix with cohesive elements. (**b**) Model of contacts at the vise interface and the matrix.

**Figure 4:**
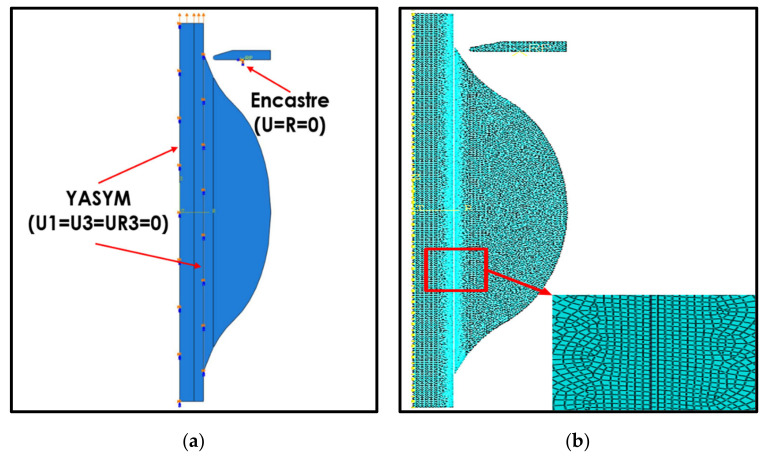
(**a**) The boundary and load conditions; (**b**) the mesh.

There were two types of contact used in this analysis. As can be seen in Figure 3a, both the fiber and the matrix shared nodes with the cohesive elements. The mesh element applied to the cohesive zone was not matched to the mesh elements of the fiber and matrix, as shown in Figure 4a. Therefore, the discretization level in the cohesive layer was different from the discretization level in the surrounding structures. As a result, tie-constraint contact was applied between the cohesive elements with the adjacent components [21]. Hence, the fiber and matrix were appointed as the master surface and the cohesive element was appointed as the slave surface. 

Secondly, the surface-to-surface contact model was applied to the interface area of the retaining vise with a binding matrix, as shown in Figure 3b. The discretization method applied to the contact model property was node-to-surface, with normal behavior used as the contact interaction property. Conversely, the type of pressure–overclosure relationship used was the “hard” contact model, which was used by setting the vise surface as the master surface and the outer surface of the binding matrix as the slave surface.

At the fiber and matrix interface, the contact model used was a cohesive element contact model. Cohesive element parameters in the cohesive zone contact model such as stiffness coefficient, initiation stress, and fracture energy (*K_nn_, t_nn_,* and *G_IC_,* respectively) were obtained by using the method developed by [15,22]. With regard to the selection of cohesive element parameters found in previous research [23], the interface shear strength values of the cohesive properties include the stiffness coefficient (K_nn_ of 2700 N/mm^3^, K_tt_ of 2700 N/mm^3^, and K_ss_ of 2700 N/mm^3^), fracture energy of 15.15 N/mm, and damage initiation (t_nn_ = 270 N/mm^2^, t_tt_ = 270 N/mm^2^, and t_ss_ = 270 N/mm^2^).

### 2.2. Selection of Boundary Condition and Load 

The provision of boundary conditions in the microbond testing model was applied to three main components, namely, the fiber, the matrix, and the vise elements. The vise was conditioned as a rigid element with a single reference point. In order to prevent movement of the vise, we applied the boundary condition of the encastre type to the reference point of the vise with the fixed support condition (U1 = U2 = UR1 = UR2 = UR3 = 0), where *Un* is the movement in the direction of the *n* axis and UR*n* is the movement of rotation in the direction of the *n* axis; as shown in Figure 4a, the vise was not allowed to move in any direction. Conversely, for the fiber and matrix elements, the boundary condition applied was antisymmetry in the Y plane (YASYMM), wherein the fiber and matrix were only allowed to move in the direction of the loading plane. The loading method was carried out with a displacement application system that was applied to the upper end of the fiber. The amount of displacement applied was the same as the magnitude of the displacement that occurred in the experimental measurement results, which was 0.466 mm in the same direction as the fiber axis. Previous research [23] has reported that the fine-mesh type with 61,016 elements is the most suitable type for testing simulations of *Typha angustifolia* fiber/epoxy microbonds according to the comparison of simulation and experimental results; this mesh is shown in Figure 4b.

### 2.3. IFSS and Critical Fiber Length Calculation 

The interfacial shear strength of the *Typha angustifilia* fiber–matrix bond was calculated using the following equation [24]:

(1) $$\tau =\frac{F_{max}}{\pi dl_{e}}$$

where *F_max_* is the maximum load when the debonding process occurs, *d* is the fiber diameter, and *l_e_* is the embedded fiber length in the matrix. 

The critical length equation is as follows [25]:

(2) $$l_{c}=\frac{\sigma d}{2\tau }$$

where *l*_c_ is the critical length, σ is the tensile strength of the fiber, *d* is the fiber diameter, and τ is the interfacial shear stress. 

## 3. Results

### 3.1. Comparison with Experimental Microbond Test

Simulations were carried out by setting the fiber diameter, embedded fiber length, and vise–specimen distance to fit the experimental microbond test carried out in a previous study [23]. The parameters are given in Table 2.

**Figure 5:**
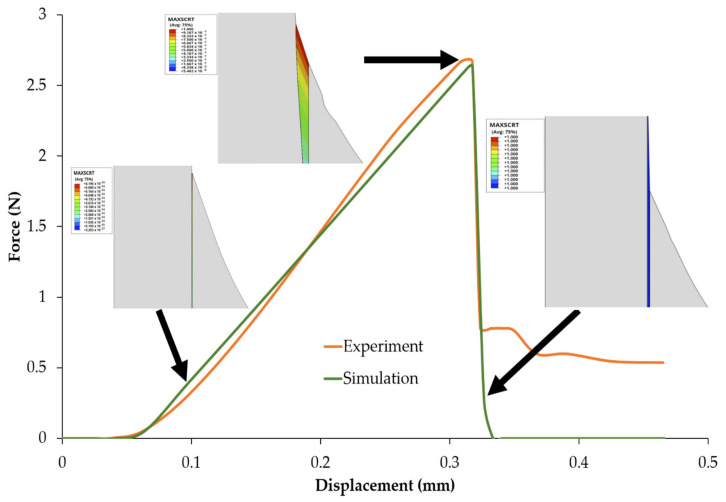
Cohesive element failure.

Figure 5 shows the simulation results along with the experimental microbond test results. It can be seen that the simulation results agreed well with the experimental microbond test. In addition, the cohesive failure during the simulation can clearly be observed. Figure 5 shows the maximum traction damage initiation criterion (MAXSCRT), a scalar parameter with a total failure of one. Lower and higher MAXSCRT values are indicated by the blue and red contours, respectively. At a displacement of 0.1 mm, the red and blue contours were valued at 6.19 × 10^−14^ and 3.25 × 10^−17^, respectively, indicating that failure at the upper cohesive layer had not yet begun. When the displacement was 0.3 mm, the upper cohesive layer began to fail as the value of the red contour reached one, whereas the value of the blue contour was 5.46 × 10^−5^. The cohesive layer failed completely after 0.3 mm displacement, as indicated by both the red and blue contour values reaching one.

Cohesive failure occurs when the force reaches a maximum value before the force decreases. This maximum value is the basis for the calculation of the IFSS, as shown, for example, in [14]. In this study, it was approximately 2.55 N for the simulation and 2.63 N for the experimental microbond test.

### 3.2. Effect of Fiber Diameter

**Figure 6:**
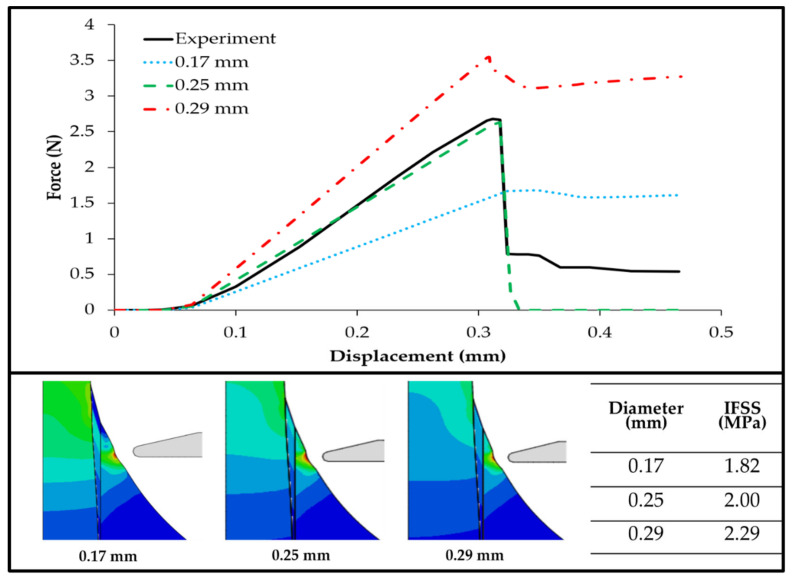
Force–displacement curve simulation and IFSS values of various fiber diameters.

Small deviations in the test parameters can have a significant impact on the test results, and this is the case for the fiber diameter effects on the IFSS value. The effect of various fiber diameters on the maximum load was assessed; three variations of fiber diameter, namely 0.17, 0.25, and 0.29 mm, were applied to the *Typha angustifolia* fiber/epoxy microbond test model. Figure 6 shows a significant difference in the maximum loads received by the fibers of varying diameters. Early on, the load–displacement curves recorded in the microbond test showed the same general tendency until the peak load value was reached, after which the fiber–matrix separation caused the load value to drop sharply. 

For the fiber with a smaller diameter, the maximum load was also smaller at 1.67 N, whereas for the fiber with a larger diameter, the maximum load was greater at 3.54 N, and for 0.25 mm diameter fibers, the maximum load received by the fiber was 2.91 N. The value of the fiber and matrix interface shear strength (IFSS) was calculated using Equation (1). Upon obtaining the maximum force and the displacement curve, we were able to calculate the IFSS value for each fiber diameter comparison model. From the calculation results, we found that the largest fiber diameter produced the highest IFSS value at 2.29 MPa, as shown in Figure 6. Conversely, for the smallest fiber diameter, the IFSS value obtained was also smaller at 1.82 MPa. Moreover, for the 0.25 mm fiber diameter, the IFSS value obtained was 2.0 MPa, with the difference from the original value being 0.04 MPa. 

On the basis of the results of the calculation of the IFSS value for each of the fiber diameters, by applying Equation (2), we then found that the critical length of the fiber for the fiber diameter of 0.17 mm was 3.57 mm. The critical lengths of the fibers for the fiber diameters of 0.25 and 0.295 mm were 4.65 and 4.80 mm, respectively, whereas the critical length of the fiber in our experimental test results was 4.57 mm. The distribution of the contact friction stress occurred at the beginning of the debonding condition. The point of high contact friction stress was found on models with larger fiber diameters. The large area of the surface in the center of the embedded length had a very low contact friction stress at the start of debonding, and as a result, the IFSS was found to be much larger for the models of a larger fiber diameter. 

### 3.3. Effect of Fiber Embedded Length

**Figure 7:**
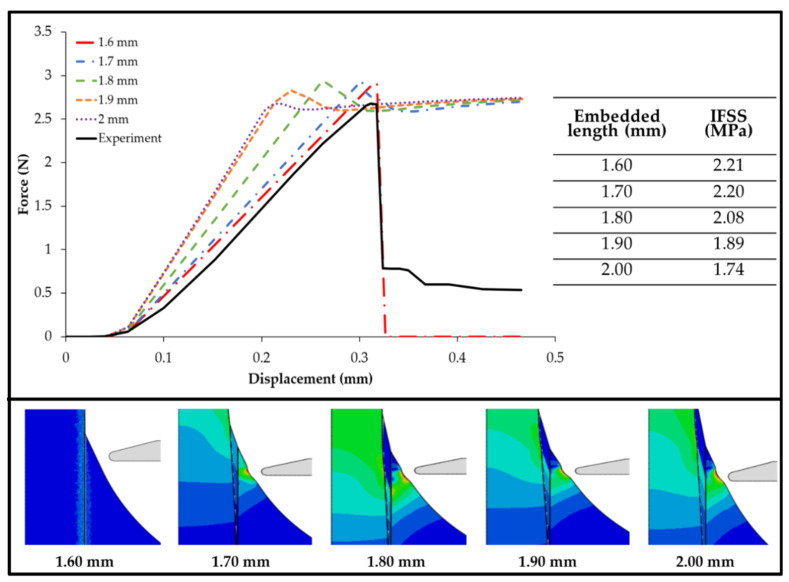
Force–displacement curve simulation and IFSS value of various embedded lengths.

The force–displacement curve simulation of embedded length variations is provided in Figure 7. The size of the embedded length was 1.6 mm in the experiment. When simulated, it showed a curve similar to the experiment, whereas the other embedded length variations did not show an alignment with the experimental results. The sizes of 1.6, 1.7, 1.8, 1.9, and 2 mm had maximum forces of 2.91, 2.86, 2.94, 2.82, and 2.65 N and displacement sizes of 0.31, 0.29, 0.26, 0.23, and 0.2 mm, respectively. The longer the embedded length, the smaller the displacement that occurred. This occurred because the area of the epoxy attached to the fiber was larger. 

The maximum force on the five models of various fiber length embedded in the matrix is also shown in Figure 7, showcasing the comparison of the IFSS value in each model, which varied from the highest value of 2.21 MPa found in the model with an embedded length of 1.6 mm. The model with an embedded length of 2.0 mm produced the smallest IFSS value of 1.74 MPa, whereas the models with embedded lengths of 1.7, 1.8, and 1.9 mm provided IFSS values of 2.20, 2.08, and 1.89 MPa, respectively. On the basis of the curve of the numerical simulation results, we were able to conclude that the length of the fiber embedded in the matrix was inversely proportional to the IFSS value between the fibers and the matrix. The longer the fiber embedded in the matrix, the smaller the IFSS value.

### 3.4. Effect of Vise–Specimen Distance

**Figure 8:**
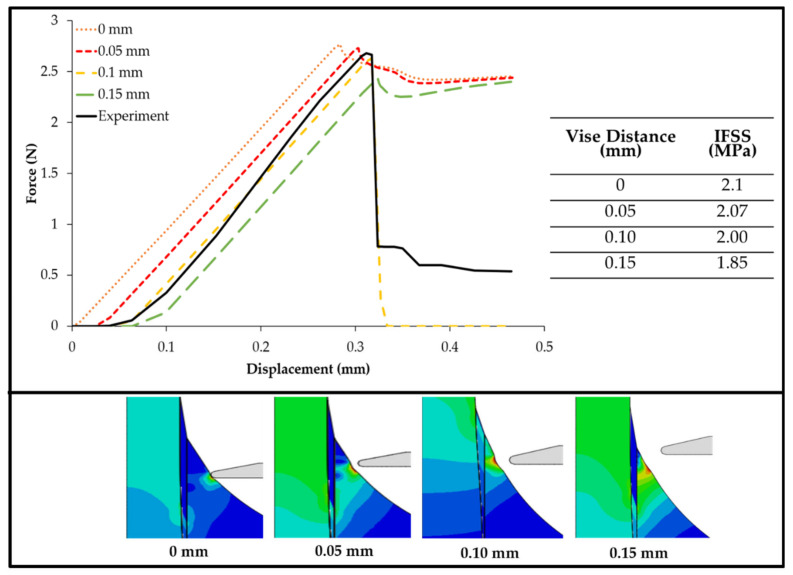
Force–displacement curve simulation and IFSS values of various vise distances from the specimen.

The numerical method for simulating the effect of vise distance on the microbond test model was applied. In the analysis of the effect of vise distance or restraint on the matrix, we found the results to be inversely proportional to the vise distance and the maximum force received by the fiber. Figure 8 shows the force–displacement curve simulation for the variation of the vise distance from the specimen; the distances of the vise to the specimen of 0, 0.05, and 0.15 mm did not show any similarity with the experimental results. When the vise distance from the specimen rose, the displacement increased as the maximum force was reduced. This occurred because when the vise moved away from the microbond test model, the effect of the vise on stress distribution was reduced, stress concentration was reduced, and loads were more effectively transferred between the fiber and the resin. The maximum force received by the fiber was found in the model with the closest distance between the specimen and the vise, the latter touching the matrix with a distance of 0 mm. In the model with the vise touching the matrix, the maximum force received by the fiber was 2.77 N, whereas the smallest maximum force occurred in the model with the farthest distance from the vise to the specimen, which was 0.15 mm, with a maximum force received by the fiber of 2.38 N. For the model with a distance of 0.05 mm between the vise and the specimen, the maximum force received by the fiber was 2.73 N, and in the models with vise distances of 0.1 and 0.15, the maximum forces received by the fiber in each model were 2.63 and 2.43 N, respectively. The vise distance of 0 mm had the highest IFSS value at 2.1 MPa, whereas the vise distance of 0.15 mm had the lowest IFSS value with a value of 1.85 MPa, as shown in Figure 8. The IFSS simulation value increased with decreasing distance between the specimen and the vise.

### 3.5. Effect of Fiber Aspect Ratio

**Figure 9:**
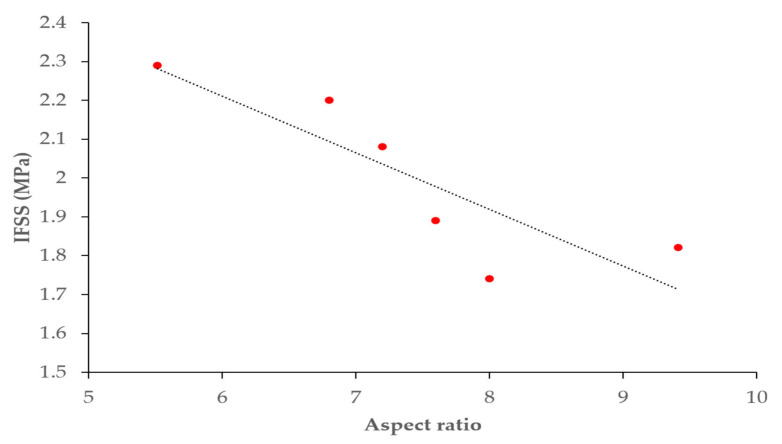
IFSS vs. embedded fiber length to fiber diameter aspect ratio.

The fiber aspect ratio is herein defined as the ratio of embedded fiber length to fiber diameter. Figure 9 shows the relationship between the fiber aspect ratio and IFSS. It can be seen that the fiber aspect ratio had an inverse relationship with IFSS. The highest value of 2.29 MPa was found for the ratio of embedded length to fiber diameter of 5.5. The aspect ratio of 8 produced the smallest IFSS value of 1.74 MPa, whereas the ratios of embedded length to fiber diameter of 9.4, 6.8, 7.2, and 7.6 mm produced IFSS values of 1.82, 2.2, 2.08, and 1.89 MPa, respectively.

## 4. Discussion

The microbond test of natural fibers is influenced by several factors that are difficult to control, such as the resin droplet size and embedded fiber length. These factors are also the sources of IFSS value scattering that is typical in a microbond test of natural fibers. Therefore, numerical simulations may provide a supplement to the experimental microbond test where these parameters can be controlled. 

In this study, numerical simulations were carried out to investigate the role of three parameters in influencing a microbond test’s results. A microbond test environment of *Typha angustifolia* fiber/epoxy was simulated using finite element analysis. A simulation was carried out to compare the results with previous studies [23]. The simulation of the microbond test of the *Typha angustifolia* fiber/epoxy showed exceptional agreement with the experimental microbond test from a previous study [18]. The simulation was carried out using the experimental parameters shown in Table 2 that were taken from a previous study [23]. The simulation captured the progression of displacement vs. force of a microbond test, including the point of cohesive failure. In this study, the maximum point of the force in the numerical simulation was determined when the force started to drop with additional displacement. This force drop indicated that cohesive failure had (partially or fully) occurred. A partial force drop indicated partial cohesive failure. The maximum point was a point taken just before the drop, despite the partial or full cohesive failure. This is the basis for the IFSS calculation used in this study, as well as in another study [14].

By using the experimental parameter values in [23] as the base values, we carried out further simulations by varying the three parameters in order to investigate the role of each parameter in influencing a microbond test’s results, as well as consequently modulating the IFSS value. The numerical simulation results showed that larger fiber diameters led to increased IFSS values in a microbond test (Figure 6). Similar results have also been reported, wherein it was found that the larger the fiber diameter, the higher the IFSS value for Typha fiber/epoxy resin [26]. This is due to the proportional relationship between fiber diameter, average peak force, and IFSS [11]. 

The numerical simulation results also indicate that a longer embedded fiber length decreases the IFSS value. In a related study, Zhi et al. [27] observed that the area ratio between the high-stress point and the contact interface area decreased with increasing embedded fiber length, consistent with the decreasing trend of IFSS. Samples with small embedded lengths had the highest overall contact friction stresses at the interface at the start of debonding. This explains why the IFSS was higher in the case of the short embedded lengths.

Another finding of this study is that increasing the vise–specimen distance leads to decreased values of IFSS in a microbond test setting. Several researchers have studied the effect of vise angles on IFSS, as determined by the microbond test [28,29]. Miller et al. [8] showed that the position of the loading blades greatly affects the yield and that the bond strength may be overestimated as the separation distance increases. Using finite element analysis (FEA), researchers found that the distribution of data observed in the experiments was due to the inability to control the shape of the loading blades and the spacing of the blades between the experiments [17]. These findings are comparable to the results found in this study. The fiber aspect ratio also has an inverse relationship with the IFSS. This is comparable to a previous study [30], wherein the relationship between the IFSS and the ratio of embedded length to the fiber diameter was significantly decreased. 

Table 3 summarizes the numerical simulation results as compared to some previous studies. The numerical simulation shows strong potential as a tool for modeling an experimental microbond test where difficult-to-control parameters can easily be tuned in a simulated environment. The role of each parameter can be concluded on the basis of the numerical simulation results. A larger fiber diameter may lead to a higher IFSS value in a microbond test, whereas increasing the vise–specimen distance and a longer embedded length may lead to a lower IFSS value in a microbond test. In a microbond test, the interaction between these three parameters may produce IFSS scattering that is typically observed. For example, a lower-than-average IFSS data point may be produced by a longer-than-average embedded fiber length in a sample or a lower-than-average fiber diameter. Hence, the modeling in this study has demonstrated that a simulated microbond test can be carried out to supplement the experimental microbond test, especially when intractable parameters are considered. 

### Limitations

In Figure 6, Figure 7 and Figure 8, one can observe that partial cohesive failures occurred during the simulation since the force drops did not reach the near-zero point. This could be attributed to the cohesive properties in the simulation and provides an opportunity for future investigation. 

## 5. Conclusions

A microbond test of natural fiber tends to produce IFSS value scattering, hindering the characterization of a composite fiber. This data scattering originates from many known experimental parameters, such as the size of a resin droplet, meniscus angle, fiber diameter, embedded fiber length, vise distance, and testing environment. In this paper, we used a numerical simulation to investigate the role of experimental parameters in IFSS value scattering of the microbond test of *Typha angustifolia* fiber/epoxy. Three parameters were selected in this study: fiber diameter, fiber length embedded in the epoxy, and the distance between the vise and the specimen. 

This study found that fiber diameter had a direct relationship with the IFSS value, whereas embedded length and vise–specimen distance had an inverse relationship with the IFSS value. These results suggest that the three parameters investigated in this study could cause a deviation in a microbond test’s results in a different way. A larger fiber diameter leads to higher IFSS values in a microbond test. On the other hand, a longer embedded length and a shorter vise–specimen distance lead to lower IFSS values in a microbond test. These findings can be used as a reference for future investigation of the sources of IFSS value scattering in a microbond test, with the eventual aim of reducing data deviation of a microbond test. 

In the future, we recommend investigating the role of other experimental parameters such as the resin droplet size and the meniscus angle. In addition, we suggest investigating the influence of cohesive properties in the numerical simulation of a microbond test. Lastly, it is advised that the role of each experimental parameter be compiled and classified in order to modulate the IFSS value. These are important steps in order to gain a comprehensive understanding of IFSS value scattering in a microbond test.

## Figures and Tables

**Table 1:** Material properties of *Typha angustifolia* fiber/epoxy resin [18,19,20].

	Young’s Modulus *E* (MPa)	Tensile Strength σ (MPa)	Poisson’s Ratio *v*
*Typha angustifolia* Fiber	884.66	74.46	0.35
Epoxy resin	3700	89.45	0.45

**Table 2:** Experimental microbond test parameters used in [23] that were also used in this study.

Parameters	Value
Fiber diameter	0.25 mm
Embedded fiber length	1.60 mm
Vise–specimen distance	0.10 mm

**Table 3:** Comparison with other models.

Observation of Current Results	Comparable with:
Larger fiber diameter led to higher IFSS value	Reference [26]
IFSS decreased with increasing embedded length	Reference [27]
Larger fiber diameter led to higher average peak force	Reference [11]
The force increased as the distance between the vise and the specimen grew	Reference [31]

## Data Availability

Not applicable.
